# Outcomes of Ambulatory General Surgery in Older Patients: A Retrospective Study

**DOI:** 10.7759/cureus.91107

**Published:** 2025-08-27

**Authors:** Eva Borges, Sara Fernandes, Catarina Spínola Almeida, Fabiana Silva, Jelena Pajic, Luís Miranda

**Affiliations:** 1 General Surgery, Unidade Local de Saúde (ULS) Santa Maria, Lisbon, PRT; 2 Faculty of Medicine, University of Lisbon, Lisbon, PRT

**Keywords:** aging, ambulatory procedures, general surgery, geriatric surgery, patient safety, surgery

## Abstract

Introduction*:* Older adults represent a growing proportion of surgical patients. Ambulatory surgery is increasingly used for its efficiency and lower costs. This study assessed its safety in individuals over 75 compared to younger cohorts.

Methods*: *A retrospective observational study was conducted in a dedicated Ambulatory Surgery Unit, including patients over 75 years of age who underwent general surgical procedures under general anesthesia from January 2020 to January 2023. Ambulatory surgery was defined as procedures requiring less than 24 hours of hospital stay, including cases with overnight observation. A control group of patients under 75 years was selected through simple random sampling. The primary outcome was surgical reintervention within 30 days. Secondary outcomes included emergency department visits and postoperative complications. Data on comorbidities, ASA classification, and postoperative morbidity were collected and analyzed. Statistical comparisons employed chi-square tests, t-tests, or ANOVA as appropriate, with a significance threshold of p < 0.05.

Results*:* A total of 405 patients were included, of whom 205 were aged over 75 and 200 were under 75. Older patients had significantly higher ASA scores (p < 0.001) and Charlson Comorbidity Index values (1.89 ± 1.6 vs. 1.37 ± 1.4; p = 0.001). Despite this, no significant differences were observed in surgical reintervention rates (1.0% vs. 2.0%; p = 0.394) or overall complication rates (17.1% vs. 15.5%; p = 0.668). Most complications in both groups were minor (Clavien-Dindo grade I-II). Unplanned emergency visits were significantly less frequent in the older cohort (1.0% vs. 4.5%; p = 0.029). Hospital stays longer than 24 hours occurred in only 1.0% of elderly patients. No differences were found in procedure type distribution between age groups.

Discussion*: *Our findings suggest that ambulatory general surgery is a safe and feasible option for patients aged over 75 when appropriate selection criteria are applied. Despite higher comorbidity and ASA scores, elderly patients experienced comparable complication and reintervention rates to younger counterparts, with significantly fewer unplanned emergency visits. These results highlight the importance of functional status and overall health rather than age alone in determining surgical eligibility.

Conclusion*: *Ambulatory surgery can be safely offered to selected elderly individuals. Comprehensive preoperative evaluation, rather than chronological age, should guide surgical decisions in this growing population.

## Introduction

The global population is undergoing a profound demographic transition, characterized by a steady increase in the proportion of older individuals. This shift has significant implications for public health systems [1]. Portugal is currently the fourth-oldest country in the world in terms of the proportion of elderly people relative to the total population [2]. According to the Portuguese National Statistics Institute, 24.1% of the population of Portugal was aged 65 or over in 2023.

As the global population continues to age, there has been a significant shift in the field of surgery. An increasing number of procedures are now being performed on older adults [3]. A study has shown that approximately 53% of all surgeries are conducted on individuals aged 65 and over, and it is estimated that nearly half of this population will undergo at least one surgical procedure during their lifetime [4]. Simultaneously, ambulatory surgery has become an increasingly prominent component of modern surgical practice, offering reduced costs, shorter recovery times and greater efficiency. However, elderly patients have unique physiological characteristics, and while some studies have addressed outpatient surgery in this population, the available literature remains limited and lacks a comprehensive evaluation of its safety.

As patients over the age of 75 are often considered to be at greater surgical risk due to age-related physiological changes, particularly frailty and functional decline, this study aimed to assess whether ambulatory surgery is as safe for this age group as it is for younger patients [5]. Therefore, this study sought to determine whether age alone affects the safety and outcomes of day surgery for this age group in our Ambulatory Unit.

## Materials and methods

A retrospective observational study was conducted on patients operated on in our Ambulatory Surgery Unit. For the purposes of this study, ambulatory surgery refers to procedures involving a hospital stay of less than 24 hours, even when this includes a single overnight stay. Our unit is specifically designated for managing these short-stay surgical patients. Eligibility for ambulatory surgery in our unit is based on clinical and social criteria. Patients must be in stable health (ASA I-III), undergo procedures expected to last less than 120 minutes with a low risk of complications, and have pain that can be managed with oral medication. Social requirements include living within 60 minutes of the hospital, understanding and accepting perioperative instructions, arranging a responsible carer for the first 24 hours after the procedure and ensuring access to telephone contact.

The inclusion criteria comprised patients aged over 75 years who underwent surgical procedures within the remit of general surgery (including abdominal wall, hepatobiliary and pancreatic, endocrine, breast and gastrointestinal tract surgeries) in our Ambulatory Surgery Unit between January 2020 and January 2023. Patients who underwent procedures under local anesthesia and/or sedation, or surgeries belonging to other surgical specialties were excluded.

The primary outcome was the need for surgical reintervention within 30 days of surgery. Secondary outcomes included unplanned visits to the emergency department, hospitalization longer than 24 hours and postoperative complications within the same 30-day period.

Data were collected from clinical records and included demographic characteristics, medical history, surgical details and postoperative outcomes. The Clavien-Dindo classification system for postoperative complications [6], the American Society of Anesthesiologists (ASA) Physical Status Classification System [7], and the Charlson Comorbidity Index (CCI) [8] were used in this study. All three tools are publicly available and do not require permission for use in academic research. A control group was formed through simple random sampling of patients under 75 years of age. Randomization was performed using a computer-generated list of random numbers, and the sample size was chosen to match that of the study group.

Categorical variables are reported as frequencies and percentages, and comparisons between groups were made using the chi-square test (χ²). Continuous variables are presented as means and standard deviations (SD), with comparisons performed using independent t-tests or analysis of variance (ANOVA), as appropriate. When the assumption of homogeneity of variances was violated (Levene’s test, p < 0.05), Welch’s test was applied. For all key outcomes, test statistics (e.g., χ² values, t values), degrees of freedom, and exact p-values were reported. Ninety-five percent confidence intervals (95% CI) were also calculated for primary and secondary outcomes where applicable. A p-value < 0.05 was considered statistically significant. Statistical analyses were performed using IBM SPSS Statistics for Windows, Version 29 (Released 2022; IBM Corp., Armonk, New York, United States). A small number of missing data were present in secondary variables, and these cases were excluded from the respective analyses but retained for all other outcomes.

Ethical disclosures

The authors declare that the procedures followed were in accordance with the regulations of the relevant clinical research ethics committee and those of the Code of Ethics of the World Medical Association (Declaration of Helsinki as revised in 2024).

## Results

Over a 36-month period, a total of 205 patients aged over 75 years were included in the study and received surgical procedures under general anesthesia, performed by the Department of General Surgery in our ambulatory surgery unit. For comparative purposes, a control group consisting of patients under 75 years who underwent surgery during the same timeframe was established using a simple randomization method. The final study cohort comprised 405 patients, most of whom were female (n = 211; 52.1%), with a mean age of 69 ± 13.1 years.

The mean age of the study group was significantly higher (78.9 ± 3.5 vs. 59.8 ± 12.1 years; t(403) = 21.63, p < 0.001; 95% CI: 17.3-21.1). The proportion of female patients was comparable between groups (54.6% vs. 49.5%; χ²(1) = 1.01, p = 0.315). The mean CCI score was also significantly higher in the older group (1.89 ± 1.6 vs. 1.37 ± 1.4; t(403) = 3.26, p = 0.001; 95% CI: 0.21-0.83).

The ASA physical status differed significantly between groups (χ²(2) = 22.8, p < 0.001), with the older group presenting a greater proportion of ASA II and III patients, indicating a greater burden of systemic disease (Table 1).

**Table 1:** Demographic and clinical characteristics of patients undergoing ambulatory surgery stratified by age group. The table includes age, sex, comorbidities (CCI), ASA classification, procedure type, hospital stay duration, complications, emergency visits, and reintervention rates.

Characteristics	All (n=405)	>75 years (n=205)	<75 years (n=200)	p-value
Gender - n (%)				0.031
male	194 (47.9)	93 (45.4)	101 (50.5)	
female	211 (52.1)	112 (54.6)	99 (49.5)	
Age - mean+-SD	69.5+-13.1	78.9+-3.5	59.8+-12.1	<0.001
ASA- n (%)				<0.001
I	77 (19.0)	46 (22.4)	31 (15.5)	
II	257 (63.5)	110 (53.7)	147 (73.5)	
III	71 (17.5)	49 (23.9)	22 (11.0)	
CCI - mean+-SD	1.63+-1.5	1.89+-1.6	1.37+-1.4	<0.001
Surgical procedure				0.153
Hospitalisation >24h – n (%)				0.171
yes	8 (2.0)	2 (1.0)	6 (3.0)	
no	397 (98.0)	203 (99.0)	194 (97.0)	
Morbidity - n (%)				
yes	66 (16.3)	35 (17.1)	31 (15.5)	0.668
no	339 (83.7)	170 (82.9)	169 (84.5)	
Emergency service - n (%)				0.029
yes	11 (2.7)	2 (1.0)	9 (4.5)	
no	394 (97.3)	203 (99.0)	191 (95.5)	
Surgical reintervention- n (%)				0.394
yes	6 (1.5)	2 (1.0)	4 (2.0)	
no	399 (98.5)	203 (99.0)	196 (98)	

The distribution of surgical procedures was not significantly different between groups (χ² = 11.74, df = 7, p = 0.153). The most frequently performed procedures in both groups were inguinal-scrotal hernia repair and total thyroidectomy, accounting for over 60% of all surgeries. Hospital stays longer than 24 hours occurred in 1.0% of elderly patients and 3.0% of younger patients (χ²(1) = 1.89, p = 0.170) (Table 2).

**Table 2:** Distribution of surgical procedures by age group in the Ambulatory Unit Frequencies and types of general surgical procedures performed in patients aged >75 and <75 years

Surgical procedure	All (n=405) n (%)	>75 years (n=205) n (%)	<75 years (n=200) n (%)
Inguinal-scrotal hernia repair	137 (33.8)	76 (37.1)	61 (30.5)
Incisional hernia repair	24 (5.9)	10 (4.9)	14 (7.0)
Umbilical hernia repair	24 (5.9)	10 (4.9)	14 (7.0)
Total thyroidectomy	122 (30.1)	68 (33.2)	54 (27.0)
Thyroid lobectomy	18 (4.4)	8 (3.9)	10 (5.0)
Subtotal parathyroidectomy	15 (3.7)	8 (3.9)	7 (3.5)
Laparoscopic cholecystectomy	21 (5.2)	11 (5.4)	10 (5.0)
Other procedures	44 (10.9)	14 (6.8)	30 (15)

Within 30 days postoperatively, the complication rate was 17.1% in the older group (95% CI: 11.9%-23.1%) and 15.5% in the younger group (95% CI: 10.3%-21.6%), with no statistically significant difference (χ²(1) = 0.18, p = 0.668). Emergency department visits were significantly less frequent in the older group (1.0% vs. 4.5%; χ²(1) = 4.74, p = 0.029), although the association was weak (Cramer’s V = 0.108; Phi = -0.108), indicating a slight inverse correlation between age and emergency visits.

Reoperation was required in 1.0% of older patients vs. 2.0% in younger patients (χ²(1) = 1.02, p = 0.394). Most complications in both groups were minor (Clavien-Dindo I or II). Only six patients (1.5%) required surgical reintervention (Table 3).

**Table 3:** Clavien-Dindo Classification of postoperative complications by age group Distribution of minor (grade I–II) and major (grade IIIa–IIIb) complications in patients who experienced postoperative morbidity

Clavien-Dindo Classification	All (n=66) n (%)	>75 years (n=35) n (%)	<75 years (n=31) n (%)
I	36 (54.5)	22 (62.9)	14 (45.2)
II	16 (24.4)	5 (14.3)	11 (35.5)
IIIa	8 (12.1)	6 (17.1)	2 (6.4)
IIIb	6 (9.0)	2 (5.7)	4 (12.9)

Among patients who required surgical reintervention, 83.3% were female, but this was not statistically significant (χ²(1) = 1.52, p = 0.218). No significant differences were observed regarding age, ASA classification, CCI, or procedure type between those who did and did not require surgical reintervention (Table 4).

**Table 4:** Comparison of clinical variables between patients with and without surgical reintervention Demographic and clinical variables were compared between patients who required surgical reintervention and those who did not. Includes sex, age, ASA, CCI, and procedure type.

Variables	Reoperation (n=6)	No reoperation (n=399)	p-value
Gender - n (%)			0.218
male	1 (16.7)	193 (48.4)	
female	5 (83.3)	206 (51.6)	
Age - mean+-SD	70.0+-4.1	69.4+-13.2	0.086
ASA- n (%)			0.361
I	0	77 (19.3)	
II	4 (66.7)	253 (63.4)	
III	2 (33.3)	69 (17.3)	
CCI - mean+-SD	1.33+-1.9	1.64+-1.5	0.313
Surgical procedure			0.800

## Discussion

The global demographic landscape is changing significantly, with a consistent rise in the number of older people. Projections suggest that, by 2050, people aged 65 and over will account for around 16% of the global population, up from 8% in 2010 [9]. Within this demographic, the subgroup of individuals aged 75 and over is growing rapidly, presenting unique challenges to healthcare systems worldwide. The World Health Organization refers to this age group as 'older adults', acknowledging the distinct health considerations associated with advanced age [10]. This shift demands a re-evaluation of current healthcare practices, particularly in surgical care, to address the specific needs and risks of this growing population segment.

Our study reflects the demographic reality of an aging population and the significant number of surgeries performed on older individuals. During the period covered by this retrospective study, approximately 3,385 adult patients underwent a general surgical procedure in our Ambulatory Unit. Of these patients, around 39% were aged over 65 years and 6% were aged over 75. These data are in line with estimates from Chia and Tan [3], who noted that over half of all surgeries are now performed on patients over 65, underscoring the relevance of surgical care in this age group, even in the context of ambulatory surgery, where patient selection is stricter.

Although numerous studies address the perioperative risks in older adults, most are framed from an anaesthesiology perspective and focus on cognitive decline, polypharmacy, and altered drug metabolism [4,11,12]. Frailty has also emerged as a crucial independent predictor of adverse outcomes [5]. Hansen et al. [13] and Charipova et al. [14] emphasize the importance of individualized preoperative assessments, including cognitive and frailty screening, particularly in ambulatory settings.

Despite these considerations, few studies have specifically assessed the safety and outcomes of general surgery in elderly patients within an ambulatory framework. Cao et al. [11] found that when appropriately selected, older patients can achieve outcomes comparable to younger patients, with similar rates of complications and readmissions. Our findings support this, showing low complication and reintervention rates in patients over 75 years, despite their higher ASA scores and comorbidities.

While some studies have investigated surgical outcomes in elderly patients, few have specifically focused on those undergoing general surgical procedures in the ambulatory setting. This model of care is increasingly adopted for its efficiency and cost-effectiveness, yet it introduces unique challenges for older adults, who are more susceptible to postoperative complications. Our study contributes to this underexplored area by focusing on elderly patients in the context of ambulatory general surgery.

The surgical reintervention rate in our study was 1.0% for elderly patients, which compares favorably with the rates reported by Chia and Tan [3], Zenilman [15], and Cao et al. [11] in both inpatient and outpatient contexts. Importantly, most reinterventions were due to postoperative bleeding, requiring a return to the operating room for hemostasis revision, and occurred after total thyroidectomy or bariatric procedures, reflecting the complexity rather than age alone as a driver of adverse outcomes.

Interestingly, older patients in our study had fewer unplanned emergency visits compared to younger patients, despite a slightly higher rate of minor complications. This aligns with observations by Hodgson et al. [16], who noted that structured postoperative follow-up and improved access to primary care in elderly patients may reduce emergency service utilization. Our results reinforce the idea that comprehensive perioperative planning and continuity of care are essential in this group.

Overall, our findings align with the perspective shared by Rajan et al. [17] and Vann [18], who advocate for expanding access to ambulatory surgery in older populations, provided that selection criteria are strict and perioperative care is optimized. Rather than chronological age, factors such as functional status, frailty, and comorbidity burden should inform surgical decision-making.

Some limitations of our study should be acknowledged. As a retrospective, single-center analysis, the generalizability of our findings may be limited. Selection bias may limit external validity, as the analysis reflects outcomes only in patients over 75 deemed appropriate for ambulatory surgery. Furthermore, we were unable to assess frailty scores or functional status due to data limitations, which are known to be strong predictors of postoperative outcomes [5,14]. Nonetheless, our use of objective clinical endpoints, inclusion of a younger control group, and real-world setting strengthen the relevance of our findings.

Future multicenter prospective studies should explore these results further, incorporating structured geriatric assessments, frailty scoring, and patient-reported outcomes. Integrating geriatric-specific protocols such as prehabilitation, early discharge planning, and personalized follow-up may further enhance the safety and effectiveness of ambulatory surgery in older adults.

## Conclusions

This study supports the safety and feasibility of ambulatory general surgery in well-selected patients aged over 75. Despite higher baseline comorbidity and ASA scores, elderly patients demonstrated low rates of surgical reintervention and unplanned emergency visits, comparable to, or in some cases better than, their younger counterparts. These findings reinforce that surgical decisions should not rely solely on chronological age but rather on individualized assessments considering comorbidities, frailty, and functional status. Ambulatory surgery, when integrated into a patient-centered perioperative approach, can be a safe and efficient option for the aging population.
